# Remedies for Chronic Diarrhœa

**Published:** 1877-04

**Authors:** 


Remedies for Chronic Diarrhœa.—Dr. J. K. Spender writes to the *British Medical Journal:*

Medical men are often troubled with cases in which a painless diarrhœa is the leading symptom. It may come and go with the changes of temperature (being specially influenced by dampness of air and decaying vegetation), or it may be the sequel of an acute form of the malady which was never properly cured during the summer. Assuming that most of the common remedies within reach have been tried, and have only partially succeeded, I venture to recommend, firstly, a systematic use of the more powerful vegetable astringents, somewhat as follows: (*a*) A teaspoonful of tincture of galls in an ounce of distilled water, three times a day, is extremely effective, and should be continued at least once daily for some weeks. (*b*) The liquid extract of bael has many merits, and may be given in the same way. (*c*) Salicin should be administered in a dose of five or six grains, perhaps combined with a grain of ipecacuanha. Let them be mixed into a couple of pills, and taken three or four times a day. This plan seldom fails to appease an obstinate diarrhœa. But, secondly, opium is now and then absolutely necessary, and I contend that it should always be prescribed in comparatively small and frequent doses, so as to obtain the least physiological with the most medicinal effect. Let the wine of opium be given to an adult in the quantity of three or fours minims (with an ounce of chloroform water) five or six times in the twenty-four hours, and the remedy ought invariably to be left off by degrees.

---

The Increase of Nervous Diseases.—Dr. Althans, in Med. Times and Gaz., London, in answer to the question, "Do the conditions of modern life favor the developement of nervous diseases?" states in an elaborate statistical paper that such is not the case. We cannot answer for England, but feel well assured that in this country nervous affections, particularly paralysis and neuralgias, are far more frequent than they were twenty-five or thirty years ago; a fact which we think is in a large degree attributable to the use of tobacco, coffee, tea and opium.—*Louisville Md. News.*

